# BMI, Waist Circumference Reference Values for Chinese School-Aged Children and Adolescents

**DOI:** 10.3390/ijerph13060589

**Published:** 2016-06-14

**Authors:** Peige Song, Xue Li, Danijela Gasevic, Ana Borges Flores, Zengli Yu

**Affiliations:** 1 School of Public Health, Xinxiang Medical University, Xinxiang 453003, China; peigesong@163.com (P.S.); xue.li@ed.ac.uk (X.L.); 2 Centre for Population Health Sciences, University of Edinburgh, Edinburgh EH8 9AG, UK; danijela.gasevic@ed.ac.uk; 3 School of Philosophy, Psychology and Language Sciences, University of Edinburgh, Edinburgh EH8 9AD, UK; ablborges@gmail.com

**Keywords:** BMI, waist circumference, childhood obesity, China

## Abstract

*Background*: Childhood obesity has become one of the most serious public health challenges in the 21st century in most developing countries. The percentile curve tool is useful for monitoring and screening obesity at population level, however, in China, no official recommendations on childhood body mass index (BMI) and waist circumference (WC) reference percentiles have been made in practice. *Aims*: to construct the percentile reference values for BMI and WC, and then to calculate the prevalence of overall and abdominal obesity for Chinese children and adolescents. *Methods*: A total of 5062 anthropometric records for children and adolescents aged from 7 to 18 years (2679 boys and 2383 girls) were included for analysis. The participants were recruited as part of the national representative “China Health and Nutrition Survey” (CHNS). Age, gender, weight, height, and WC were assessed. Smoothed BMI and WC percentile curves and values for the 3rd, 5th, 10th, 15th, 25th, 50th, 75th, 85th, 90th, 95th and 97th percentiles were constructed by using the Lambda-Mu-Sigma (LMS) method. The prevalence estimates of the overall and abdominal obesity were calculated by using the cut-offs from our CHNS study and the previous “Chinese National Survey on Students’ Constitution and Health” (CNSSCH) study, respectively. The difference between prevalence estimates was tested by a McNemar test, and the agreement between these prevalence estimates was calculated by using the Cohen’s kappa coefficient. *Results*: The prevalence values of overall obesity based on the cut-offs from CHNS and CNSSCH studies were at an almost perfect agreement level in boys (κ = 0.93). However, among girls, the overall obesity prevalence differed between the studies (*p* < 0.001) and the agreement was weaker (κ = 0.76). The abdominal obesity prevalence estimates were significant different according to the two systems both in boys and girls, although the agreement reached to 0.88, which represented an almost perfect agreement level. *Conclusions*: This study provided new BMI and WC percentile curves and reference values for Chinese children and adolescents aged 7–18 years, which can be adopted in future researches. Large longitudinal study is still needed to reveal the childhood growth pattern and validate the inconsistence between different percentile studies.

## 1. Introduction

Childhood obesity, defined as an excessive fat accumulation status in children [1,2], has become one of the most serious public health challenges in the 21st century [3]. Globally, approximately 170 million children (aged less than 18 years) are suffering from the physical and psychological consequences caused by overweight or obesity [4,5], which may result in decreased life quality and increased risk of developing non-communicable diseases such as cardiovascular disease, type 2 diabetes and cancers in their later adulthood life [6,7]. Although there are still many regions struggling with child hunger, such as South-eastern Asia and Sub-Saharan Africa [8], the childhood obesity has already reached endemic proportions by extending to developing countries, including China [9,10].

At the population level, percentile curve is a useful tool for monitoring and screening obesity among school-aged children and adolescents [11]. For measuring overall obesity, the most commonly used indicator is Body Mass Index (BMI) [12]. Generally, overweight and obesity in children are defined respectively based on the 85th percentile and 95th percentile of BMI among children [13]. Although BMI has been used to demonstrate the weight-for-height relationship and to some extent can provide an estimate of the body fat percentage, BMI alone cannot reflect the fat distribution very comprehensively, such as the abdominal fat deposits [14].

Abdominal obesity, which is the deposition of visceral fat, is associated with higher risk of metabolic complications, such as hyperlipidaemia, and diabetes [15,16]. Commonly adopted predictive indicators of abdominal obesity include waist circumference (WC) and related indices such as the waist-to-height and waist-to-hip ratios [16,17,18]. WC is a relatively simple and convenient measure and can be readily used to estimate the accumulation of abdominal fat [19]. Given that children are growing and their bodies are developing over the years, a uniform referent value may not be appropriate. Previous literates suggest the use of age- and gender-specific WC ≥ 90th percentile as the threshold for abdominal obesity [18,20,21].

In China, no official recommendations on childhood BMI and WC reference percentiles have been made in clinical or population practice, however, based on different investigations, several reference percentiles have been established at both local and national levels [22,23,24,25,26]. Among them, the most nationally representative BMI and WC percentiles were generated based on a large cross-sectional school-based study conducted in 2005, namely, the “Chinese National Survey on Students’ Constitution and Health” (CNSSCH) [24,27], which participants included students from primary and high schools aged 7–18 years [28]. The main limitation of the study was that the percentiles were established based on only cross-sectional data, this may therefore limit the reflection of the real BMI and WC growth pattern with age [24,27]. In addition, the childhood growth pattern changes with time, economic and social development, so it needs to be regularly updated [29,30], the current CNSSCH BMI and WC reference percentiles were based on data in the year of 2005, the usage may be implausible.

Another prospective household-based study “China Health and Nutrition Survey” (CHNS), which has been conducted successively in the years of 1989, 1991, 1993, 1997, 2000, 2004, 2006, 2009 and 2011 covering nine provinces (Guangxi, Guizhou, Heilongjiang, Henan, Hubei, Hunan, Jiangsu, Liaoning and Shandong) with different geographies, economic development levels, and health indicators [31,32], has both the merits of national representativeness and longitudinal follow-up quality which allows better reflection of growth pattern [31]. To the best of our knowledge, no percentile curves for BMI and WC have been established based on the data from CHNS for school-aged children and adolescents (7–18 years old) in China. In addition, although the CNSSCH BMI and WC percentiles have made inter-national comparisons with the percentiles proposed by WHO and the US Center for Disease Control and Prevention, it is still imperative to address the previous study limitations and describe percentiles based on a new national dataset and then conduct an intra-national comparison.

In this study, we aim to construct the percentile reference values for BMI and WC based on CHNS data, and then to calculate the prevalence of overall and abdominal obesity by using CHNS and CNSSCH cut-offs and make an intra-national comparison.

## 2. Materials and Methods

### 2.1. Study Sample

Detailed information on the CHNS study design has been published elsewhere [31]. The study procedures were approved by the Institutional Review Boards of the University of North Carolina, Chapel Hill and the Chinese Centre for Disease Control [33]. This study is a secondary data analysis using public and de-identified dataset obtained from the most recent four CHNS (2004, 2006, 2009 and 2011) to construct the percentile values, no ethics approval was needed [34]. A total of 3262 school-aged participants were included in the analysis, among them, 100 (3.07%) participants had the complete four follow-ups from 2004 to 2011, 314 (9.63%) were followed for three times, 872 (26.73%) were followed for two times, and 1976 (60.58%) were only investigated for once, not all the participants were enrolled in the first survey in 2004, new participants were gradually added in the following surveys, the average timed measurement was 1.55 for one participant. Large sample size is essential for constructing percentile values at each age group [35], so we included all the anthropometric records in these four CHNS and adopted the mixed cross-sectional/longitudinal sample. Finally, a total of 5062 anthropometric records (2679 boys and 2383 girls) were included and assessed for age, gender, weight, height, and WC.

### 2.2. Anthropometry

Standard procedures were followed to conduct anthropometric measurements by well-trained examiners. Weight was measured to the nearest 0.1 kg in light clothing by using the calibrated beam scale. Height was measured to the nearest 0.1 cm without shoes by using a portable stadiometer, and WC was measured at a midpoint between the lowest rib and the iliac crest in a horizontal plane by using non-elastic tape [33,36]. All data collection staff were trained in an interobserver reliability testing before surveys [37]. BMI was calculated as weight (kg) divided by height squared (m^2^).

### 2.3. Statistical Analysis

General characteristics of the children and adolescents were reported as mean ± SD or as counts and percentages. Gender-specific percentile values were calculated and smoothed by the Lambda-Mu-Sigma (LMS) method with the LMS Chart Maker Pro version 2.3 software (The Institute of Child Health, London, UK) [38], which summarizes a changing percentile at every age point based on Box–Cox power transformation by three smoothed curves: L (lambda, skewness), M (mu, median) and S (sigma, coefficient of variation). We presented the following percentile curves for BMI and WC: 3rd, 5th, 10th, 15th, 25th, 50th, 75th, 85th, 90th, 95th and 97th. The 5th, 50th and 95th percentiles of BMI, and the 10th, 50th and 90th percentiles of WC were plotted to compare between boys and girls and with the corresponding values in the CNSSCH references. Overall obesity was defined as having age- and gender-specific BMI percentile ≥95th and abdominal obesity was defined as having age- and gender-specific WC percentile ≥90th. The prevalence of obesity was determined for the CHNS study subjects by adopting the cut-offs of the CHNS and CNSSCH classification systems, respectively, and McNemar test was used to compare the differences in prevalence. The agreement between these prevalence was tested by the Cohen’s kappa coefficient (κ). The agreement of κ < 0.00 indicates “poor”, 0.00 ≤ κ ≤ 0.02 “slight”, 0.21 ≤ κ ≤ 0.40 “fair”, 0.41 ≤ κ ≤ 0.60 “moderate”, 0.61 ≤ κ ≤ 0.80 “substantial” and 0.81 ≤ κ ≤ 1.00 “almost perfect” agreement [39,40,41]. All statistical analyses were conducted in IBM SPSS Statistics 19. Statistical significance was considered at *p* < 0.05 and all tests were two-sided.

## 3. Results

**Figure 1:**
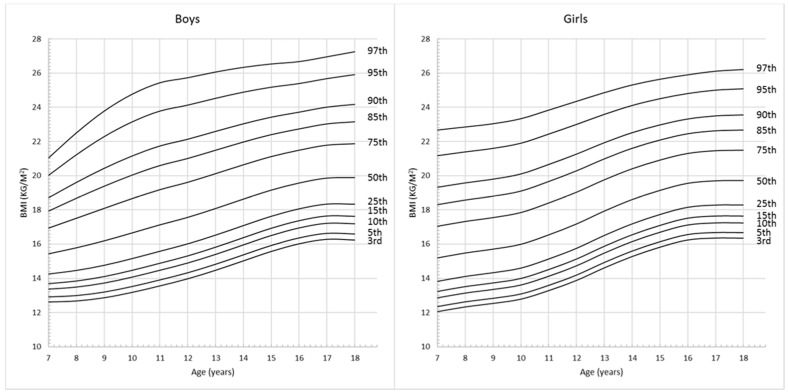
Gender- and age- specific BMI percentile curves for Chinese children and adolescents aged 7–18 years.

**Figure 2:**
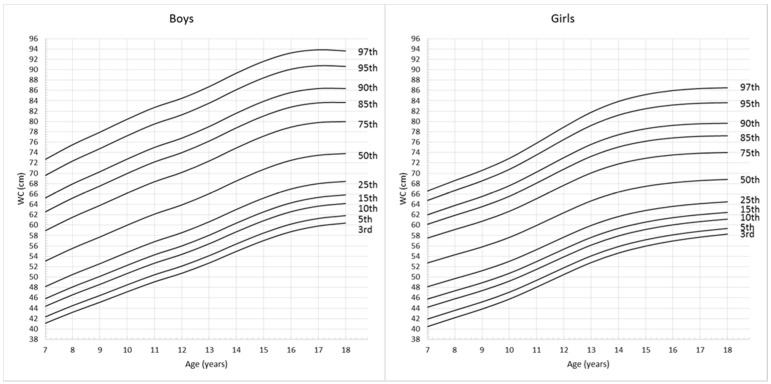
Gender- and age- specific WC percentile curves for Chinese children and adolescents aged 7–18 years.

The descriptive characteristics of the anthropometric records are shown in Table 1. Among all the 5062 records for school-aged children and adolescents, 2679 (52.9%) were boys and 2383 (47.1%) were girls. The average BMI were 18.2 ± 3.33 kg/m^2^ in boys and 17.9 ± 3.24 kg/m^2^ in girls, the average WC were 64.9 ± 10.89 cm in boys and 62.2 ± 9.42 cm in girls. Figure 1 and Figure 2 show the full set of smoothed percentile curves of BMI and WC respectively for Chinese children and adolescents aged from 7 to 18 years according to gender. The corresponding 3rd, 5th, 10th, 15th, 25th, 50th, 75th, 85th, 90th, 95th and 97th percentile values for BMI and WC are presented in Table 2 and Table 3.

**Figure 3:**
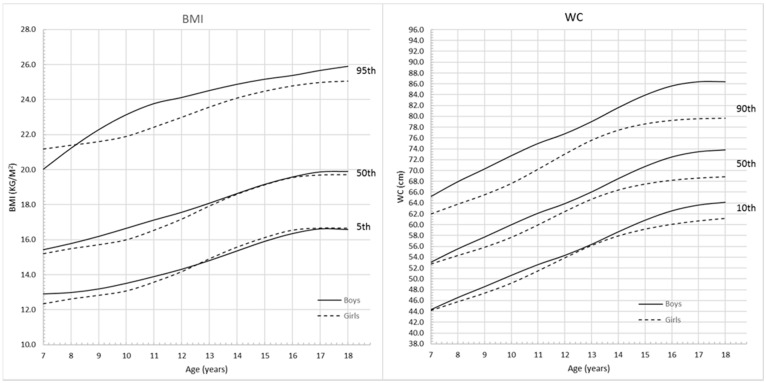
Comparison of the BMI and WC percentile curves for Chinese boys and girls aged 7–18 years.

Figure 3 shows the comparison of BMI and WC percentiles between boys and girls respectively. For BMI curves, before the age of 8, the upper 95th percentile values were higher for girls than boys. Between the ages of 8 and 12.5 years, 5th, 50th and 95th percentiles for boys remained above those for girls. After the age of 12.5 years, the lower 5th percentile values for girls were higher than those for boys. However, from 17 years, the 5th and 50th BMI percentile values were similar for both boys and girls. WC for boys and girls increased with age, and 10th, 50th and 90th percentile values for boys were higher than those for girls. The curves started to be flat from 17 years for boys and 15 years for girls, and the overall absolute increase in WC was greater among boys than girls.

**Figure 4:**
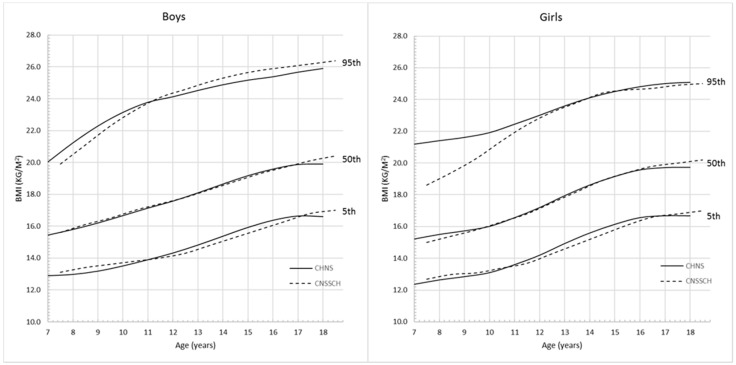
Comparison of the CHNS and CNSSCH BMI percentile curves for Chinese boys and girls aged 7–18 years.

**Figure 5:**
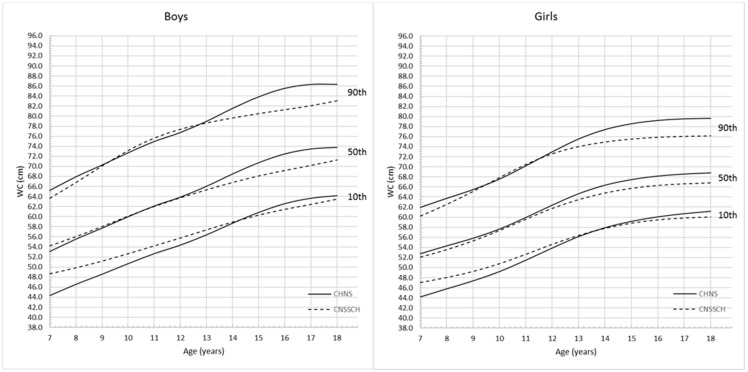
Comparison of the CHNS and CNSSCH WC percentile curves for Chinese boys and girls aged 7–18 years.

Figure 4 and Figure 5 show the comparison of the 5th, 50th and 95th percentile curves based on CHNS and CNSSCH data. For BMI (Figure 4), the CHNS and CNSSCH percentiles showed a similar shift in distribution; however, among boys, the CHNS-based percentiles started to flatten from 17 years while CNSSCH-based curves continued with increasing trend after 17 years. 

Among girls, the CHNS-based upper 5th percentile values were higher than those derived from CNSSCH data, and this trend was more apparent before the age of 12 years. For WC (Figure 5), the increasing patterns of the CHNS and CNSSCH percentiles were slightly different. Among boys, the CHNS percentiles showed a rapid growth pattern before 17 years and then started to flatten while the CNSSCH-based curves showed a continuous increasing trend across ages. Among girls, the difference of WC changing patterns was not very obvious, both the CHNS- and CNSSCH-based percentile curves started to flatten from the age of 15 years, and values for CNSSCH were lower at all 10th, 50th and 90th percentiles.

The prevalence values of overall obesity calculated by the CHNS and CNSSCH cut-offs are shown in Table 4. The overall obesity prevalence was 5.7% for males aged 7–18 years according to the CHNS reference, which was slightly lower than that calculated based on the CNSSCH reference (5.8%). There was no significant difference (*p* > 0.05) between the prevalence, and the coefficient of agreement κ was 0.93, which represented an almost perfect agreement level. Among girls aged 7–18 years, the overall obesity prevalence was 5.0% according to the CHNS reference, which was significantly lower (*p* < 0.001) than that according to the CNSSCH reference (7.7%). The coefficient of agreement κ was only 0.76, which represented a substantial agreement level.

The prevalence of abdominal obesity calculated by the CHNS and CNSSCH cut-offs were shown in Table 5, the abdominal obesity prevalence was 9.9% for male children and adolescents aged 7–18 years according to the CHNS reference, which was significantly lower (*p* < 0.001) than that according to the CNSSCH reference (11.8%). The coefficient of agreement κ was 0.88, which represented an almost perfect agreement. 

Among girls aged 7–18 years, the overall obesity prevalence was 10.2% according to the CHNS reference, which was also significantly lower (*p* < 0.001) compared to that based on the CNSSCH reference (12.5%), although the agreement was also almost perfect (κ = 0.88).

## 4. Discussion

In this study, we constructed the BMI and WC reference values for Chinese children and adolescents aged 7–18 years based on a large national representative study. To the best of our knowledge, this is the most up-to date national BMI and WC reference for school-aged children and adolescents in China. The proposed BMI and WC reference values can be adopted as useful references for monitoring both overall and abdominal obesity among Chinese children and adolescents.

The current widely adopted BMI and WC reference values for identifying obesity among Chinese school-aged children and adolescents were based on the CNSSCH [24,27]. Its large sample size can guarantee the study preciseness in generating reference values, but the cross-sectional study design may limit its use to reflect how BMI and WC change with age [24]. In this study, the notably difference of BMI percentile curves was in CHNS reference curves, the BMI started to be flat before 18 years but the CNSSCH curve still showed increasing trend at 18 years, which was also observed among girls. In addition, the CHNS WC growth pattern also started to reach plateau before adulthood both among boys and girls, while the CNSSCH WC curve showed the potential to keep the increasing trend after 18 years among boys. This difference may be explained by the data property of these two studies, the mixed cross-sectional/longitudinal may have a better performance in reflecting the real growth pattern.

BMI and WC are widely used to define obesity at population level [13,21]. Generally, both the overall and abdominal obesity prevalence were overestimated by the CNSSCH cut-offs, this may because that the CNSSCH reference was only based on anthropometric data of children and adolescents aged 7–18 years at the year of 2005, while the CHNS percentile curves were established by using more up-to-date data from 2004 to 2011. These overall obesity prevalence estimates for boys were in good agreement with the CNSSCH study, however, the difference of overall obesity among girls were larger, which needs future validation. The abdominal obesity prevalence identified by these two reference systems were in high agreement both among boys and girls but still significantly different. In addition, the comparison between the prevalence of obesity based on CHNS and CNSSCH revealed that CNSSCH might overestimate the prevalence of obesity, which should be of caution in the future application. Both CHNS and CNSSCH reference systems have merits, the former was derived from mixed cross-sectional/longitudinal data, but the latter had a larger sample size. Since the absence of national BMI and WC percentile curves calculated by large longitudinal studies in Chinese or even Asian children and adolescents, it’s hard to conclude the best performing system in identifying obesity prevalence among school-aged children and adolescents, the difference between these two systems also reveals the urgent needs to build new reference values of BMI and WC by large national cohort study.

However, the CHNS reference derived in this study may still be imperfect because it’s not a pure longitudinal study, and the sample size at the age of 18 years was not enough (less than 100), this might also influence the accuracy of our reference estimation. Despite the limitations, this study has several strengths: first of all, this study is a national representative survey, the generated CHNS percentile curves can be used at the national level; secondly, the CHNS percentiles curves were based on both cross-sectional and longitudinal data which will better reflect the growth pattern during childhood.

## 5. Conclusions

To conclude, our study has provided new national BMI and WC percentile curves and reference values for Chinese children and adolescents aged 7–18 years, since our findings were based on a mixed cross-sectional/longitudinal database, the proposed percentiles should be considered as reference but not as the real growth pattern standard in future practice.

## Figures and Tables

**Table 1:** Characteristics of the anthropometric records (*n* = 5062, mean (SD)).

Age	Boys (*n* = 2679)			Girls (*n* = 2383)		
	*n* (%)	BMI (kg/m^2^)	WC (cm)	*n* (%)	BMI (kg/m^2^)	WC (cm)
7 (7.0–7.9)	261 (9.74)	15.8 (2.44)	54.1 (7.33)	203 (8.52)	16.0 (3.71)	52.9 (6.30)
8 (8.0–8.9)	223 (8.32)	16.4 (3.00)	57.0 (7.96)	203 (8.52)	16.1 (2.93)	54.9 (6.76)
9 (9.0–9.9)	236 (8.81)	16.8 (3.30)	58.4 (7.55)	241 (10.11)	16.3 (3.11)	56.1 (7.22)
10 (10.0–10.9)	257 (9.59)	17.2 (3.15)	60.9 (8.57)	219 (9.19)	16.5 (2.85)	57.9 (7.62)
11 (11.0–11.9)	278 (10.38)	18.0 (3.36)	63.8 (9.29)	239 (10.03)	17.2 (2.98)	60.8 (8.17)
12 (12.0–12.9)	235 (8.77)	17.9 (2.89)	64.2 (8.92)	231 (9.69)	17.7 (3.00)	63.1 (8.46)
13 (13.0–13.9)	243 (9.07)	18.8 (3.24)	66.9 (8.82)	211 (8.85)	18.4 (2.58)	65.4 (7.05)
14 (14.0–14.9)	231 (8.62)	19.1 (2.96)	69.7 (9.09)	274 (11.50)	19.1 (2.66)	67.3 (8.10)
15 (15.0–15.9)	227 (8.47)	19.8 (3.14)	71.9 (10.25)	186 (7.81)	19.6 (2.70)	68.3 (7.97)
16 (16.0–16.9)	212 (7.91)	19.9 (2.62)	73.3 (8.34)	176 (7.39)	19.9 (2.25)	68.7 (6.88)
17 (17.0–17.9)	181 (6.76)	20.4 (2.63)	74.6 (8.88)	132 (5.54)	20.2 (2.76)	69.6 (7.49)
18 (18.0–18.9)	95 (3.55)	20.3 (2.98)	74.2 (9.42)	68 (2.85)	20.0 (2.35)	69.8 (7.96)
Total (7.0–18.9)	2679 (100.00)	18.2 (3.33)	64.9 (10.89)	2383 (100.00)	17.9 (3.24)	62.2 (9.42)

**Table 2:** Gender- and Age- specific BMI percentile values for Chinese children and adolescents aged 7–18 years (*n* = 5062).

Age	Percentiles for Boys											Percentiles for Girls										
	3rd	5th	10th	15th	25th	50th	75th	85th	90th	95th	97th	3rd	5th	10th	15th	25th	50th	75th	85th	90th	95th	97th
7	12.6	12.9	13.4	13.7	14.2	15.4	16.9	17.9	18.7	20.0	21.0	12.1	12.4	12.9	13.2	13.8	15.2	17.0	18.3	19.3	21.2	22.7
8	12.7	13.0	13.5	13.9	14.5	15.8	17.5	18.7	19.6	21.2	22.5	12.3	12.6	13.1	13.5	14.1	15.5	17.3	18.6	19.6	21.4	22.9
9	12.9	13.2	13.7	14.1	14.8	16.2	18.1	19.4	20.4	22.3	23.8	12.5	12.8	13.3	13.7	14.3	15.7	17.5	18.8	19.8	21.6	23.1
10	13.2	13.5	14.1	14.5	15.1	16.7	18.7	20.0	21.2	23.1	24.8	12.8	13.1	13.6	14.0	14.6	16.0	17.8	19.1	20.1	21.9	23.3
11	13.6	13.9	14.5	14.9	15.6	17.1	19.2	20.6	21.7	23.8	25.4	13.3	13.6	14.1	14.5	15.1	16.6	18.4	19.7	20.7	22.4	23.8
12	14.0	14.3	14.9	15.3	16.0	17.6	19.6	21.0	22.1	24.1	25.7	13.9	14.2	14.7	15.1	15.8	17.2	19.0	20.3	21.3	23.0	24.4
13	14.5	14.8	15.4	15.8	16.5	18.1	20.1	21.5	22.6	24.5	26.1	14.6	14.9	15.5	15.9	16.5	17.9	19.8	21.0	21.9	23.6	24.9
14	15.0	15.4	16.0	16.4	17.1	18.6	20.6	22.0	23.0	24.9	26.3	15.3	15.6	16.1	16.5	17.2	18.6	20.4	21.6	22.5	24.1	25.3
15	15.6	15.9	16.5	16.9	17.6	19.2	21.1	22.4	23.4	25.2	26.5	15.8	16.1	16.7	17.1	17.7	19.1	20.9	22.1	23.0	24.5	25.7
16	16.0	16.4	16.9	17.4	18.1	19.6	21.5	22.7	23.7	25.4	26.7	16.2	16.6	17.1	17.5	18.2	19.6	21.3	22.4	23.3	24.8	25.9
17	16.3	16.6	17.2	17.7	18.3	19.9	21.8	23.0	24.0	25.7	26.9	16.3	16.7	17.2	17.6	18.3	19.7	21.5	22.6	23.5	25.0	26.1
18	16.2	16.6	17.2	17.6	18.3	19.9	21.9	23.2	24.2	25.9	27.2	16.3	16.7	17.2	17.6	18.3	19.7	21.5	22.7	23.6	25.1	26.2

**Table 3:** Gender- and Age- specific WC percentile values for Chinese children and adolescents aged 7–18 years (*n* = 5062).

Age	Percentiles for Boys											Percentiles for Girls										
	3rd	5th	10th	15th	25th	50th	75th	85th	90th	95th	97th	3rd	5th	10th	15th	25th	50th	75th	85th	90th	95th	97th
7	41.1	42.3	44.4	45.8	48.2	53.1	58.9	62.6	65.2	69.6	72.7	40.5	41.9	44.2	45.8	48.1	52.7	57.5	60.2	62.0	64.8	66.6
8	43.2	44.5	46.6	48.1	50.5	55.5	61.5	65.2	67.9	72.4	75.5	42.2	43.6	45.8	47.4	49.7	54.3	59.2	61.9	63.8	66.7	68.6
9	45.2	46.5	48.6	50.1	52.6	57.7	63.8	67.5	70.3	74.8	77.9	43.8	45.2	47.4	48.9	51.2	55.8	60.7	63.6	65.5	68.5	70.5
10	47.2	48.5	50.7	52.3	54.8	60.0	66.2	70.0	72.7	77.2	80.4	45.8	47.1	49.2	50.7	53.0	57.6	62.7	65.6	67.6	70.7	72.9
11	49.1	50.4	52.7	54.3	56.8	62.1	68.4	72.2	75.0	79.5	82.7	48.1	49.4	51.5	53.0	55.3	59.9	65.1	68.1	70.2	73.5	75.8
12	50.7	52.1	54.4	56.0	58.5	63.9	70.2	74.0	76.8	81.3	84.5	50.5	51.8	53.9	55.4	57.7	62.4	67.7	70.8	73.0	76.5	78.9
13	52.7	54.1	56.4	58.0	60.6	66.0	72.4	76.2	79.0	83.5	86.7	52.8	54.1	56.2	57.6	59.9	64.7	70.1	73.3	75.6	79.2	81.8
14	54.9	56.4	58.7	60.4	63.0	68.5	74.9	78.8	81.6	86.1	89.3	54.6	55.9	57.9	59.4	61.7	66.4	71.8	75.1	77.4	81.2	83.9
15	57.0	58.5	60.9	62.5	65.2	70.7	77.2	81.1	83.9	88.4	91.6	56.0	57.2	59.2	60.6	62.8	67.5	72.9	76.2	78.6	82.5	85.2
16	58.7	60.2	62.6	64.3	67.0	72.5	78.9	82.8	85.6	90.1	93.2	57.0	58.1	60.1	61.4	63.6	68.2	73.5	76.8	79.3	83.2	86.0
17	59.8	61.3	63.7	65.3	68.0	73.5	79.8	83.6	86.4	90.8	93.8	57.7	58.8	60.7	62.0	64.1	68.6	73.9	77.1	79.6	83.5	86.4
18	60.4	61.8	64.2	65.8	68.4	73.8	80.0	83.7	86.4	90.6	93.6	58.3	59.4	61.2	62.4	64.5	68.8	74.0	77.2	79.7	83.6	86.5

**Table 4:** Prevalence of overall obesity among Chinese boys and girls aged 7–18 years and the comparison based on the CHNS and CNSSCH cut-offs.

Gender	Prevalence of Overall Obesity (%)			Agreement of Prevalence by Different Cut-offs	
	CHNS	CNSSCH	*p* Value	κ	Agreement
Boys					
7	5.4	5.4	1.000	1.00 **	almost perfect
8	7.6	9.0	0.250	0.91 **	almost perfect
9	7.2	8.9	0.125	0.89 **	almost perfect
10	5.1	5.4	1.000	0.88 **	almost perfect
11	7.6	8.3	0.625	0.90 **	almost perfect
12	5.5	5.1	1.000	0.96 **	almost perfect
13	5.8	5.3	1.000	0.96 **	almost perfect
14	4.3	4.3	1.000	1.00 **	almost perfect
15	6.2	5.3	0.500	0.92 **	almost perfect
16	3.3	2.8	1.000	0.92 **	almost perfect
17	3.9	3.3	1.000	0.92 **	almost perfect
18	5.3	4.2	1.000	0.88 **	almost perfect
Total (7–18)	5.7	5.8	0.664	0.93 **	almost perfect
Girls					
7	7.4	14.3	<0.001 *	0.65 **	substantial
8	6.4	13.8	<0.001 *	0.60 **	moderate
9	5.4	11.2	<0.001 *	0.62 **	substantial
10	4.6	10.0	<0.001 *	0.60 **	moderate
11	4.2	6.3	0.063	0.79 **	substantial
12	6.9	7.8	0.625	0.87 **	almost perfect
13	3.8	3.8	1.000	1.00 **	almost perfect
14	4.7	5.1	1.000	0.96 **	almost perfect
15	3.8	4.3	1.000	0.93 **	almost perfect
16	3.4	3.4	1.000	1.00 **	almost perfect
17	4.5	6.1	0.500	0.85 **	almost perfect
18	1.5	1.5	1.000	1.00 **	almost perfect
Total (7–18)	5.0	7.7	<0.001 *	0.76 **	substantial
Both genders					
7	6.3	9.3	<0.001 *	0.79 **	substantial
8	7.0	11.3	<0.001 *	0.75 **	substantial
9	6.3	10.1	<0.001 *	0.75 **	substantial
10	4.8	7.6	0.001 *	0.73 **	substantial
11	6.0	7.4	0.039 *	0.86 **	almost perfect
12	6.2	6.4	1.000	0.91 **	almost perfect
13	4.8	4.6	1.000	0.98 **	almost perfect
14	4.6	4.8	1.000	0.98 **	almost perfect
15	5.1	4.8	1.000	0.92 **	almost perfect
16	3.4	3.1	1.000	0.96 **	almost perfect
17	4.2	4.5	1.000	0.88 **	almost perfect
18	3.7	3.1	1.000	0.91 **	almost perfect
Total (7–18)	5.3	6.7	<0.001 *	0.84 **	almost perfect

Notes: * Difference of prevalence significant (*p* < 0.05); ** Kappa coefficient significant (*p* < 0.0001).

**Table 5:** Prevalence of abdominal obesity among Chinese boys and girls aged 7–18 years and the comparison based on the CHNS and CNSSCH cut-offs.

Gender	Prevalence of Overall Obesity (%)			Agreement (κ) of Prevalence by Different Cut-offs	
	CHNS	CNSSCH	*p* Value	κ	Agreement
Boys					
7	3.8	7.3	0.004 *	0.67 **	substantial
8	11.7	12.6	0.500	0.96 **	almost perfect
9	5.9	6.4	1.000	0.96 **	almost perfect
10	8.9	8.2	0.500	0.95 **	almost perfect
11	13.7	12.6	0.250	0.95 **	almost perfect
12	10.6	10.6	1.000	1.00 **	almost perfect
13	11.5	11.5	1.000	1.00 **	almost perfect
14	11.3	14.3	0.016 *	0.86 **	almost perfect
15	12.3	16.3	0.004 *	0.84 **	almost perfect
16	9.9	14.2	0.004 *	0.80 **	substantial
17	8.8	16.6	<0.001 *	0.66 **	substantial
18	9.5	14.7	0.063	0.75 **	substantial
Total (7–18)	9.9	11.8	<0.001 *	0.88 **	almost perfect
Girls					
7	9.4	10.8	0.250	0.92 **	almost perfect
8	10.8	12.8	0.125	0.91 **	almost perfect
9	9.1	10.4	0.250	0.93 **	almost perfect
10	11.0	11.0	1.000	1.00 **	almost perfect
11	12.6	11.3	0.250	0.94 **	almost perfect
12	12.1	12.6	1.000	0.98 **	almost perfect
13	7.6	7.6	1.000	1.00 **	almost perfect
14	10.2	16.4	<0.001 *	0.73 **	Substantial
15	11.3	14.5	0.031 *	0.86 **	almost perfect
16	5.7	13.1	<0.001 *	0.57 **	moderate
17	10.6	15.9	0.016 *	0.77 **	Substantial
18	14.7	19.1	0.025	0.84 **	almost perfect
Total (7–18)	10.2	12.5	<0.001 *	0.88 **	almost perfect
Both genders					
7	6.3	8.8	<0.001 *	0.82 **	almost perfect
8	11.3	12.7	0.031 *	0.93 **	almost perfect
9	7.5	8.4	0.125	0.94 **	almost perfect
10	9.9	9.5	0.500	0.98 **	almost perfect
11	13.2	12.0	0.031 *	0.95 **	almost perfect
12	11.4	11.6	1.000	0.99 **	almost perfect
13	9.7	9.7	1.000	1.00 **	almost perfect
14	10.7	15.4	<0.001 *	0.79 **	substantial
15	11.9	15.5	<0.001 *	0.85 **	almost perfect
16	8.0	13.7	<0.001 *	0.71 **	substantial
17	9.6	16.3	<0.001 *	0.71 **	substantial
18	11.7	16.6	0.008 *	0.80 **	substantial
Total (7–18)	10.0	12.1	<0.001 *	0.88 **	almost perfect

Notes: * Difference of prevalence significant (*p* < 0.05); ** Kappa coefficient significant (*p* < 0.0001).

## Abbreviations

The following abbreviations are used in this manuscript:

BMI	Body Mass Index
WC	Waist Circumference
CHNS	China Health and Nutrition Survey
LMS	Lambda-Mu-Sigma
CNSSCH	Chinese National Survey on Students’ Constitution and Health
